# Accuracy of Landmark-guided Glenohumeral Joint Injections as Assessed by Ultrasound in Anterior Shoulder Dislocations

**DOI:** 10.5811/westjem.2021.3.50266

**Published:** 2021-11-05

**Authors:** Talib Omer, Michael Perez, Kristen Berona, Chun Nok Lam, Dana Sajed, Caroline Brandon, Jeffrey Falkenstein, Tarina Kang, Thomas Mailhot

**Affiliations:** Keck School of Medicine of the University of Southern California, Department of Emergency Medicine, Los Angeles, California

## Abstract

**Introduction:**

To determine the accuracy of landmark-guided shoulder joint injections (LGI) with point-of-care ultrasound for patients with anterior shoulder dislocations.

**Methods:**

Patients with anterior shoulder dislocations who underwent LGI were enrolled at our tertiary-care and trauma center. LGI attempts were recorded by an ultrasound fellowship-trained ED physician who determined if they were placed successfully. Pain and satisfaction scores were recorded.

**Results:**

A total of 34 patients with anterior shoulder dislocation and their treating ED physicians were enrolled. 41.1% of all LGI were determined to be misplaced (n=14). Patients with successful LGI had a greater decrease in mean pain scores post-LGI.

**Conclusions:**

LGI had a substantial failure rate in our study. Using ultrasound-guidance to assist intra-articular injections may increase its accuracy and thus reduce pain and the need for subsequent procedural sedation.

## INTRODUCTION

### Background

The shoulder joint is the most commonly dislocated joint and accounts for more than 70,000 emergency department (ED) visits per year in the United States alone. Current evidence suggests that intra-articular injection of the shoulder with local anesthetic agents can provide adequate analgesia to facilitate reduction and obviate the need for more resource-intensive methods such as procedural sedation. However, studies have not determined the rate at which landmark-guided shoulder joint injections (LGI) truly deposit local anesthetic into the joint space. Failure to deliver anesthetic into the joint space may increase complications and the need for additional analgesia and sedation. In the current study, we used point-of-care ultrasound to determine the accuracy of LGI.

### Importance

Shoulder dislocations are the most common joint injury treated in the ED, with anterior glenohumeral dislocation accounting for 95–97% of dislocations.^1^ In most institutions, the preferred method for providing the necessary pain relief and muscle relaxation to facilitate reduction involves procedural sedation and analgesia (PSA), typically with a combination of opioids and benzodiazepines.^2^ Although often effective, PSA can be time and resource intensive, requiring close monitoring by medical personnel due to the risk for severe complications such as central nervous system and respiratory depression.^3^ In light of this, the current literature suggests that intra-articular injections of the shoulder with local anesthetic can be an effective alternative to PSA for providing analgesia during reduction,^4^–^9^ especially in patients who cannot tolerate sedation.^5^,^10^ However, the use of local anesthetics assumes that these injections can be given with great accuracy.

Prior studies have relied on the palpation of anatomical landmarks to determine the point of entry for intra-articular injection.^5^,^8^,^10^ Since these studies did not use ultrasound or other imaging techniques to guide their injections, and dislocation results in significantly disrupted shoulder anatomy, it is unclear whether the local anesthetic was truly deposited intra-articularly. Several studies have reported limitations in assessing the overall effectiveness of intra-articular injections as an alternative to PSA due to the difficulty in determining the accuracy of LGI and the inconsistency of hematoma aspiration.^5^,^11^–^13^ Anecdotal experience suggests that aspiration of a hematoma from the shoulder joint prior to injection of local anesthetic is not a reliable determinant of correct intra-articular placement, with one study indicating the aspiration of blood even when the needle was in the wrong position.^14^ To date, none of the literature has evaluated the accuracy of LGI for treatment of acute anterior shoulder dislocation.

### Goals of this Investigation

The purpose of our study was to assess the accuracy of LGI for the treatment of patients with anterior shoulder dislocations. Our hypothesis was that many LGIs are not intra-articular and are, therefore, ineffective. We also evaluated the overall effectiveness of intra-articular injections as an alternative to PSA and the application of using ultrasound in the treatment of shoulder dislocations.

## METHODS

### Study Design and Setting

We conducted a prospective, observational study on a convenience sample of patients who presented to the LAC+USC ED, an urban tertiary care and trauma center. The study was approved by the USC Health Sciences Institutional Review Board.

### Selection of Participants

Patients with anterior glenohumeral shoulder dislocation diagnosed by radiography were enrolled between November 2015–October 2018. Adult patients (age > 18 years) were eligible for enrollment if the treating emergency physician decided to perform a landmark-based, intra-articular shoulder injection as part of the patient’s treatment. We excluded patients who had a shoulder fracture, inferior glenohumeral dislocation, or posterior dislocation confirmed via radiograph. Patients who had a prior history of shoulder joint replacement or contraindication to the shoulder injection, such as overlying cellulitis or allergy to lidocaine, were also excluded. Patient comprehension of the study, the potential risks, and its difference from the standard medical care were verbally assessed. All patients provided informed written consent to participate in the study

**Population Health Research Capsule**

What do we already know about this issue?

*No prior studies have evaluated the success rate of landmark-guided joint injections (LGI) for anterior shoulder dislocations*

What was the research question?

*Our goal was to assess the accuracy of landmark-guided shoulder joint injections in anterior shoulder dislocations using point-of-care ultrasound.*

What was the major finding of the study?

*Over 40% of LGI were not successful, resulting in higher pain scores compared to the successful group.*

How does this improve population health?

*Using ultrasound-guidance to assist with intra-articular lidocaine injection in anterior shoulder dislocations may result in reduction of pain.*

### Injection Technique

For each emergency physician (EP) performing LGI in this study, we recorded his or her prior experience with shoulder injections. Immediately prior to the LGI attempt, the treating EP was given the opportunity to review an illustration of the standard intra-articular injection technique for an anterior shoulder dislocation. Injections were performed using sterile technique with an 18- or 20-gauge spinal needle (total length = 8.75 centimeters), and an injection volume of 15 milliliters of 1% lidocaine without epinephrine. The EPs performing the injection were blinded to any ultrasound images obtained throughout the procedure and were not informed of the needle tip position prior to injection. After the procedure, the EPs were asked to indicate their level of comfort with the LGI attempt.

### Ultrasound Technique

Placing a curvilinear transducer C60 (FUJIFILM SonoSite, Inc, Bothell, WA) in a posterior axial position on the shoulder, an ultrasound-trained EP observed the LGI needle entering the skin in real time and acquired video clips of the procedure from the time of needle entry until needle removal. The screen of the ultrasound machine was hidden from the clinicican’s view so that they were blinded to the ultrasound-determined location of the needle. The procedure was considered successful if the needle tip was visualized within the joint space at the time of lidocaine injection.

Patients were also blinded to the success or failure of the procedure. The patient could not learn about the success of the procedure from the sonographer who was behind a screen, and the patient was further instructed not to reveal anything to the clinician.

### Physicians

The EP’s decision to perform an injection was based on their clinical decision-making and personal preference, as well as the “culture” of the department where this injection was routinely done for shoulder dislocations. None of the participating EPs had specialty training in ultrasound. Residents usually performed the injections; in a few cases an attending EP performed the procedure when no residents were in the department.

### Measurements

Before and after the intra-articular injection, the patient’s pain scores were recorded and quantified via subjective pain scale. The patient had no indication of the success/failure of placement, which might have affected their pain score. We also recorded the number of procedural sedations, the patient’s length of stay and time to discharge, the amount of parenteral pain medication administrations, and patient satisfaction scores. Additionally, the EP’s past shoulder-injection experience and comfort level were recorded prior to administration of the LGI. Post treatment, we also recorded the clinician’s likelihood of using ultrasound for future shoulder injections.

## RESULTS

We enrolled 34 patients with anterior shoulder dislocations and their treating EPs between November 2015–February 2018. The majority of patients in both the successful LGI placement and misplaced groups were male and had a history of prior dislocation in the same joint before the study encounter (Table 1). Of the 34 LGIs, 14 (41.1%) were visualized outside the joint space and determined to be misplaced. The EPs in both the successful and misplaced groups reported similar comfort levels with LGI on a five-point Likert-type scale (U = 0.5). However, there was a significant difference in the number of prior injections between the successfully placed and misplaced injection groups, with the misplaced group reporting a mean number of 5.8 prior injections compared to 1.4 in the successful group (Table 1).

Patients with successful and unsuccessful relocation were comparable in age (mean 49.6; 42.7) and first- time dislocation (mean 30.0; 35.7). However, patients with unsuccessful relocation were more likely to have a right-sided laterality compared to patients with successful relocation (64.3% vs 45%).

Pain scores before the procedure were not significantly different in both groups (*P* = 0.2), nor were pain scores significantly different afterward (*P* = 0.4). However, the successful LGI group had a significantly greater decrease in pain score of 3.8 (95% confidence interval [CI], −5.1 to −2.5) compared to a decrease of 1.9 (95% CI, −3.4 to − 0.5) for the misplaced group (*P* = 0.05). Patients in both the successful and misplaced groups received similar rates of enteral, intramuscular, or intravascular analgesics prior to LGI (*P* = 0.7). Patient satisfaction scores (4.8 success [CI, 4.2–5.3] vs 4 misplaced [CI, 3.2–4.8]) were similar, regardless of success of the LGI (*P* = 0.09).

Ultimately, 42.7% of the misplaced group required a procedural sedation for reduction (n = 6) while 45% of the successful group also required procedural sedation (n = 9, *P* = 0.9). However, three of the successful LGI cases that underwent procedural sedation required subsequent reduction attempts by orthopedic surgery due to technically challenging reductions, one of which ultimately required surgical intervention. Overall satisfaction with treatment was not significantly different between the LGI groups. Those who underwent a procedural sedation rated their satisfaction lower (3.9; CI, 3.0–4.8) than those who did not (4.8; CI, 4.6–5.1) (*P* = 0.02).

## DISCUSSION

Our results confirm substantial rates of misplaced anesthesia with the landmark-based approach and less reduction in pain in anterior shoulder dislocations. Although the current literature suggests LGI is a viable alternative to the traditional PSA, these studies did not assess the accuracy of injection. Misplaced injections fail to deliver local anesthetic into the joint space and may lead to increased pain from damaging adjacent structures.^15^ Moreover, our results show that while accurately placed LGI result in a greater decrease in pain score when compared to misplaced injections, the pain score was not significantly lower.

Other studies have examined the effectiveness of successfully placed glenohumeral joint injections. Despite successful injection, nearly half the patients in our study needed to undergo procedural sedation, which may have been a result of several outliers in the success group that ultimately required more than one procedural sedation to reduce the shoulder joint. Ultrasound guidance can be used to confirm that the needle is accurately positioned within the joint. Ultrasound also provides several advantages of being readily available, portable, and associated with few to no side effects.^16^ Conversely, intra-articular lidocaine injections (IAL) without ultrasound guidance have been associated with several potential complications that will be addressed.

Existing studies have recommended the use of IAL as a safe, effective, time-efficient alternative to PSA for providing analgesia during reduction of shoulder dislocation.^4^–^8^ Both a 2012 Cochrane systematic review^7^ of five randomized controlled trials (RCT) and 211 patients and a 2008 review of six RCTs^6^ and 283 patients found that there was no significant difference in immediate shoulder reduction success rate or pain experienced between patients placed into IAL and PSA treatment groups. Additionally, several studies found IAL to be associated with lower complication rates compared to PSA, by directly targeting the source of pain and avoiding the systemic side effects of intravenous (IV) medications.^7^,^17^,^18^ Since IAL typically does not require monitoring of oxygen saturation, electrocardiography, or IV access, it has also been associated with a significantly shorter length of stay in the ED compared to PSA,^7^,^10^,^19^ with one study finding a mean ED hospitalization time of the PSA group to be nearly four times that of the IAL group (8.1 hours vs 2.2 hours).^11^ Additionally, several studies found lidocaine injections to be less costly than PSA per visit.^11^,^10^ Miller et al^19^ noted that the cost of IV sedation was $97.64 compared with only $0.52 for use of intra-articular lidocaine per patient, although costs can vary considerably between hospitals.

Although uncommon, possible complications of using IAL include the risks for infection and chondrolysis.^4^,^20^,^21^ Despite this potential risk, none of the previously mentioned studies indicated any cases of joint infection after injection, with the rate of septic arthritis estimated to be as low as 1 in 10,000 or 1 in 50,000 injections.^22^–^24^ In 2011 Piper et al^25^ conducted a review of the use of local anesthetics and determined that long exposures, such as with the use of pain pumps, can lead to chondrolysis of human articular cartilage in vitro. However, several recent studies testing isolated human articular chondrocytes determined that 1% lidocaine delivered by pain pumps for periods of 24, 48, and 72 hours did not lead to any significant chondrolysis.^26^–^28^ Thus, the use of a single intra-articular 1% lidocaine injection is likely a safe alternative to PSA with a low risk of infection or chondrolysis.

Image-guided injections have been associated with substantially greater accuracy than LGI in both cadavers and live patients. A 2014 study by Patel et al^16^ found that there was a significantly higher success rate for ultrasound-guided shoulder injections compared with LGI in cadavers (92.5% vs 72.5%, n = 80, *P* = 0.02). Additionally, a systematic review by Daley et al^29^ determined that imaging for injections in the glenohumeral joint of live patients via ultrasound, fluoroscopy, and magnetic resonance imaging, was associated with a success rate of 95% vs 79% of injections without imaging (n = 810, *P* < 0.001). However, these studies involved non-dislocated shoulders, making it difficult to assess the effectiveness of using imaging to guide IAL injections after shoulder dislocation.

Ultrasound may be an effective application in the treatment of anterior shoulder dislocations due to its ability to provide both real-time guidance for injections and immediate diagnostic imaging.^30^ Using ultrasound guidance to assist with IAL injection may increase its accuracy, making it a more attractive alternative to PSA for providing adequate analgesia to facilitate shoulder reductions.^4^,^31^ Further studies are needed to compare clinical outcomes of patients receiving ultrasound-guided shoulder injections with those receiving LGI, ideally in a clinical RCT.

## LIMITATIONS

There are limitations to this study. First, our sample size was relatively small. There were difficulties in recruiting patients due to the infrequency of encountering anterior shoulder dislocations that met the study’s specific inclusion and exclusion criteria. Additionally, an ultrasound fellowship-trained EP had to be available during subject enrollment to sonographically record the injection. Additionally, this was a convenience sample of patients who were aware of the experiment, which may have biased their interpretation of pain to fulfill the expectations of the treating physicians. Furthermore, some of the patients received pain medications before treatment with LGI, which may have influenced their perception of pain before and after LGI.

Our study population was a specific sample of patients from Los Angeles County, who likely have different characteristics including body mass index (BMI) compared to the general population, and limits the study’s applicability to other groups. Palpation of anatomical landmarks to determine the point of entry for LGI injection may be more difficult in patients with a higher BMI and may influence the accuracy of injection. The BMI data on study participants was not available to assess its impact on the accuracy of LGI injections.

Several outcomes are difficult to explain with the available data. We suspect that procedural sedation patients were less satisfied due to length of stay; however, the available data does not include complete satisfaction data. We do know that patients requiring procedural sedation had longer lengths of stay (615.20 minutes; standard deviation [SD] 328.6) compared to those who did not require sedation (211.92. minutes; 371.57 SD). On the other hand, patients with successful placements had comparable length of stay (452.31 minutes; SD 270.08) to patients with unsuccessful placements (465.06 minutes; SD 304.37).

Although the EPs in this study may have a level of expertise with shoulder injections that is not representative of physicians from institutions elsewhere in the US, experience alone seems not to be sufficient to ensure a high rate of success without confirmation of accuracy of the injection. Lack of experience with shoulder injections has been cited as one of the reasons that most EPs currently prefer PSA over LGI for shoulder reduction.^6^ Since PSA is used more frequently for procedures in the ED, EPs are usually more proficient and comfortable with that method.^13^ The small sample size, coupled with the fact that all physicians participating in the study were under 45 years of age, allowed for little variation in physician age and years in practice to evaluate the impact on procedure quality. Physician overconfidence was not assessed as a factor that might explain worse performance.

The focus on the study was to determine the failure rate, and we did not collect relevant information about the reason for the failure. Forty-one percent of patients had misplaced anesthesia with the LGI approach, but no systematic data was collected to explain this outcome. No previous literature has examined the outcomes of this procedure. Our study was also limited in its lack of data on patient obesity or factors affecting the surgery such as difficult shoulder landmark and inadequate needle length. We did observe a recurrent error in which the needle was either placed too far posterior to the joint or in some cases was not inserted deep enough. One attending physician in the experienced group (with more than 30 prior rejections) missed, which may have skewed results in favor of the less experienced providers.

Finally, the study did not record time since dislocation or distinguish between acute traumatic, first-time anterior shoulder dislocations and recurrent dislocations. This may have influenced the treating EP’s decision to use LGI over PSA and the effectiveness of LGI as a treatment method. A patient’s prior experience with shoulder dislocations may increase or attenuate the impact of perceived pain for shoulder reduction when compared to someone with no history of prior dislocation.^12^ Anecdotal experience suggests recurrent dislocations should be easier to reduce. However, our sample size was not sufficiently powered for this subgroup analysis.

## CONCLUSION

We found a substantial failure rate of landmark-guided shoulder joint injection. Using ultrasound guidance to assist intra-articular injections may increase its accuracy, thus reducing complications and the need for subsequent procedural sedation. Further research is needed to compare clinical outcomes in patients receiving ultrasound-guided shoulder joint injections with those receiving LGI. Additional areas to explore include whether successful joint injections can decrease length of stay and improve patient satisfaction.

## Figures and Tables

**Table 1:** Emergency physician measures on landmark-guided joint injections and patient measures on pain.

	Mean (95% CI)			

	Success (n = 20)	Misplaced (n = 14)	*P*-value	U-value
EP prior Injections	1.4 (0.4 to 2.3)	5.8 (0.5 to 11.7)	0.08	.05
EP comfort level with LGI (five-point Likert scale)	3 (2.4 to 3.6)	3 (2.3 to 3.7)	1.0	
Patient pain pre-injection	9.2 (8.6 to 9.9)	8.5 (7.3 to 9.7)	0.2	
Patient pain score post-injection	5.6 (4.1 to 7.0)	6.6 (4.5 to 8.7)	0.4	
Difference in patient pain score	−3.8 (−5.1 to −2.5)	−1.9 (−3.4 to − 0.5)	0.05	

*CI*, confidence interval; *EP*, emergency physician; *LGI*, landmark-guided joint injections.
